# Supplementary material for: Contribution of Protonation to the Dielectric Relaxation Arising from Bacteriopheophytin Reductions in the Photosynthetic Reaction Centers of Rhodobacter sphaeroides
Source: Biomolecules. 2024 Oct 27;14(11):1367. doi: 10.3390/biom14111367 (PMC11591870; doi:10.3390/biom14111367)
Supplement: Supplementary file 1 [file biomolecules-14-01367-s001.zip › biomolecules-3249293-supplementary.pdf]

biomolecules

MDPI

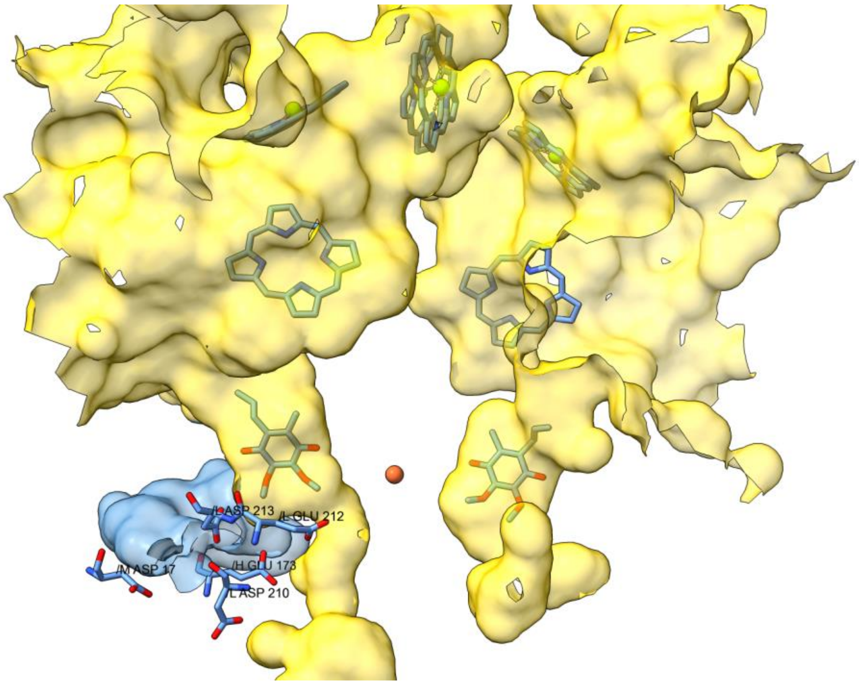

Figure S1. Identification of surface cavities in the *Rhodobacter sphaeroides* reaction center (PDB ID: 3I4D) using CASTp [80]. Surface cavities were mapped using the CASTp server to reveal solvent-accessible pockets in the protein structure. Cavities surrounding key functional components, including the RC cofactors and the acidic residues proximal to the secondary quinone acceptor (Q_B). The cavities surrounding BChl, BPheo and Q_A, Q_B are shown in gold, while the cavity near the acidic groups is depicted in blue.

CASTp tool [80] was used to analyse the solvent-accessible cavities of the reaction center (RC) of *Rhodobacter sphaeroides* (PDB ID: 3I4D), and ChimeraX [6] was applied for visualization. The focus was set on cavities around key cofactors involved in electron transport in the protein, particularly on bacteriopheophytin (I_A), primary (Q_A) and secondary (Q_B) quinones and the set of acidic groups around Q_B. CASTp revealed well-defined network of cavities providing insight into the structural features that may influence the functional dynamics of the RC.

The RC cofactors are located within an extended and connected system of cavities with a surface area of 5141.619 Å² and a volume of 8066.076 Å³. This cavity may contribute to the regulation of efficiency of the electron transfer.

Another smaller but also significant cavity includes the acidic amino acids around the secondary quinone acceptor. The cluster consists of GLU L212, ASP L213, ASP L210, GLU H173 and ASP M17, which are important for energy stabilisation and proton delivery during quinone reduction. This cavity has an area of 280.017 Å² and a volume of 105.250 Å³.

The presence of connected cavities around key cofactors highlights the importance of surface topography in modulating/controlling electron and proton transfer in the RC.

The cavity data were taken from this site:

http://sts.bioe.uic.edu/castp/index.html?3i4d

and were implemented into the ChimeraX:

https://rbvi.github.io/chimerax-recipes/castp/castp.html

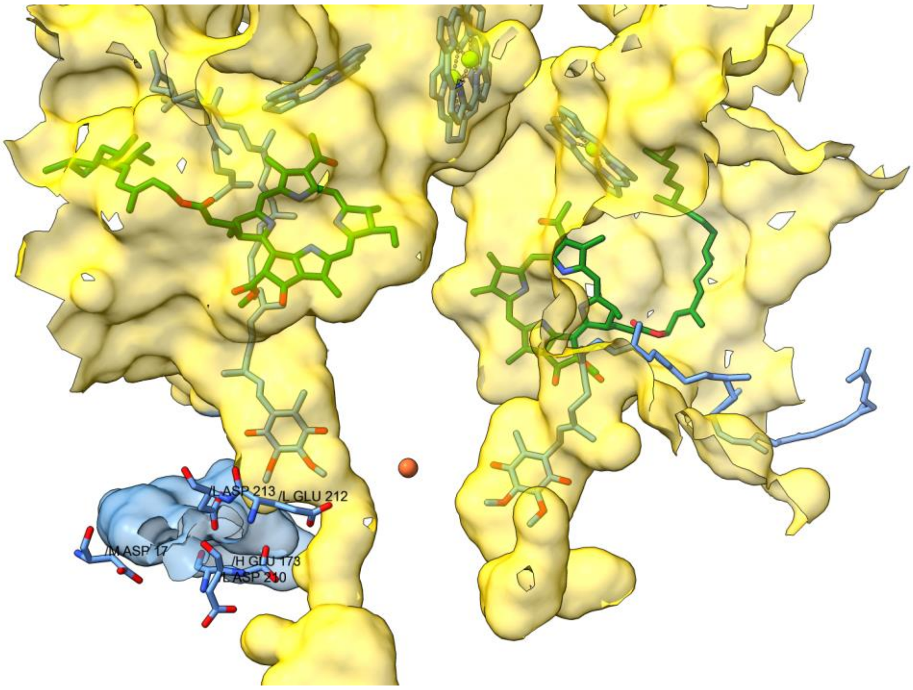

Figure S2. The system of interconnected cavities of the RC where the phytol chain of the bacteriopheophytin and isoprenoid tail of the quinone cofactors are also shown. For details see Figure. S1.

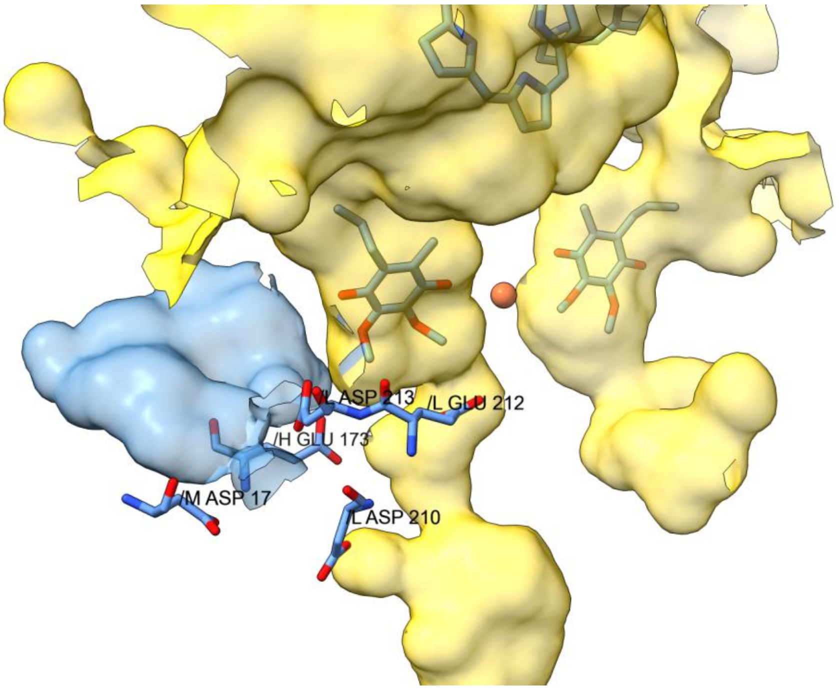

Figure S3. Close up view of the cavities around QB (gold) and the acidic cluster (blue) of the RC. For more details see Figure. S1.

3D views of the cavities (video)

Video S1. Identification of surface cavities in the *Rhodobacter sphaeroides* reaction center (PDB ID: 3I4D) using CASTp (Tian et al 2018). Surface cavities were mapped using the CASTp server to reveal solvent-accessible pockets in the protein structure. Cavities surrounding key functional components, including the RC cofactors and the acidic residues proximal to the secondary quinone acceptor (Q_B). The cavities surrounding BChl, BPheo and Q_A, Q_B are shown in gold, while the cavity near the acidic groups is depicted in green and smaller cavities at the surface in green and red. The subunits are shown by transparent colours.

Video S2. The same as in **Video S1** without subunit surfaces.

Video S3. The same as in **Video S2** with protein backbone but without subunit surfaces.

Video S4. Replacement of native UQ_{10} for AQ in Q_A binding pocket of RC from Rba. sphaeroides. The ligand binding interactions were modelled using AutoDock Vina with the 3I4D pdb protein structure as the target and AQ as the ligand. The calculations were carried out with the same positions of the amino acids as in the native structure. According to the energy states of the substituted planar three-ring structure of the AQ, several (here 4) possible arrangements with different orientations and shifts of system can be distinguished. The minimum binding energy belongs to the position depicted by blue.

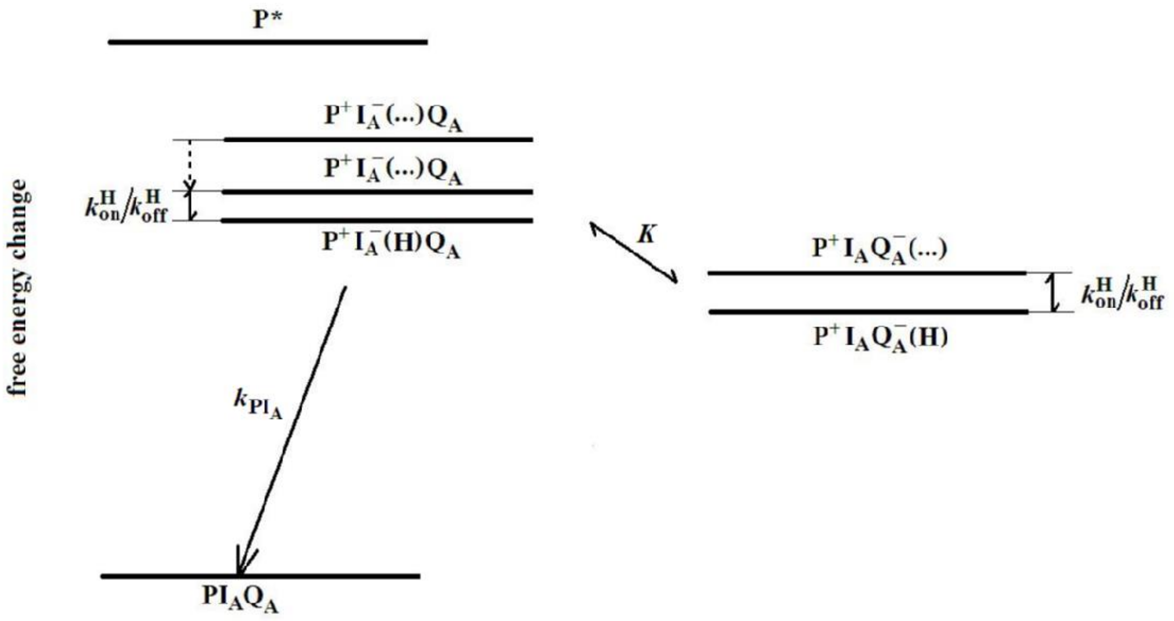

Figure S4. The free energy levels of the different redox and protonation states of the flash-induced RC relative to that of the excited dimer P*. The stabilization of the primary charge pairs $P^+I_A^-$ and $P^+Q_A^-$ occurs via dielectric relaxation (dashed line) and protonation (konH and koffH are the rate constants of proton binding and unbinding, respectively). If the native quinone UQ_{10} at the primary quinone binding site Q_A is substituted by different low potential anthraquinone (AQ) derivatives, the charge recombination $P^+I_AQ_A^- \rightarrow PI_AQ_A$ will proceed via thermally excited state $P^+I_A^-Q_A$ with apparent rate constant of k_{PI} /(1+K), where K = [$P^+I_AQ_A^-$]/[$P^+I_A^-Q_A$] is the equilibrium constant between states $P^+I_AQ_A^-$ and $P^+I_A^-Q_A$.
